# Ursodeoxycholic acid: Effects on hepatic unfolded protein response, apoptosis and oxidative stress in morbidly obese patients

**DOI:** 10.1111/liv.13562

**Published:** 2017-09-18

**Authors:** Michaela Mueller, Rui E. Castro, Anders Thorell, Hanns‐Ulrich Marschall, Nicole Auer, Merima Herac, Cecilia M.P. Rodrigues, Michael Trauner

**Affiliations:** ^1^ Hans Popper Laboratory of Molecular Hepatology Division of Gastroenterology and Hepatology Department of Internal Medicine III Medical University of Vienna Vienna Austria; ^2^ Research Institute for Medicines (iMed.ULisboa) Faculty of Pharmacy Universidade de Lisboa Lisbon Portugal; ^3^ Department of Biochemistry and Human Biology Faculty of Pharmacy Universidade de Lisboa Lisbon Portugal; ^4^ Department of Clinical Science at Danderyds Hospital Karolinska Institutet Stockholm Sweden; ^5^ Department of Surgery Ersta Hospital Stockholm Sweden; ^6^ Department of Molecular and Clinical Medicine Sahlgrenska Academy Institute of Medicine University of Gothenburg Gothenburg Sweden; ^7^ Department of Clinical Pathology University of Vienna Vienna Austria

**Keywords:** CHOP, ER stress, microRNA signalling, miR‐34a, NASH

## Abstract

**Background & Aims:**

Ursodeoxycholic acid (UDCA) is a secondary hydrophilic bile acid (BA) used as therapy for a range of hepatobiliary diseases. Its efficacy in non‐alcoholic fatty liver disease (NAFLD) is still under debate. Here, we aimed to decipher molecular mechanisms of UDCA in regulating endoplasmic reticulum (ER) homeostasis, apoptosis and oxidative stress in morbidly obese patients.

**Methods:**

In this randomized controlled pharmacodynamic study, liver and serum samples from 40 well‐matched morbidly obese NAFLD‐patients were analysed. Patients received UDCA (20 mg/kg/d) or no treatment 3 weeks before samples were obtained during bariatric surgery.

**Results:**

Patients treated with UDCA displayed higher scoring of steatosis (S), activity (A) and fibrosis (F), the so called SAF‐scoring. UDCA partially disrupted ER homeostasis by inducing the expression of the ER stress markers *CHOP* and *GRP78*. However, *ERDJ4* and *sXBP1* levels were unaffected. Enhanced *CHOP* expression, a suggested pro‐apoptotic trigger, failed to induce apoptosis via *BAK* and *BAX* in the UDCA treated group. Potentially pro‐apoptotic miR‐34a was reduced in the vesicle‐free fraction in serum but not in liver after UDCA treatment. Thiobarbituric acid reactive substances, 4‐hydroxynonenal and mRNA levels of several oxidative stress indicators remained unchanged after UDCA treatment.

**Conclusion:**

Our data suggest that UDCA treatment has ambivalent effects in NAFLD patients. While increased SAF‐scores and elevated *CHOP* levels may be disadvantageous in the UDCA treated cohort, UDCA's cytoprotective properties potentially changed the apoptotic threshold as reflected by absent induction of pro‐apoptotic triggers. UDCA treatment failed to improve the oxidative stress status in NAFLD patients.

**Abbreviations**
4‐HNE: 4‐hydroxynonenal
BA: bile acid
ER: endoplasmic reticulum
FXR: farnesoid‐X receptor
GPX: glutathione peroxidase
IR: insulin resistance
miR: micro‐RNA
mRNA: messenger ribonucleic acid
NAFLD: non‐alcoholic fatty liver disease
NASH: non‐alcoholic steatohepatitis
qRT‐PCR: quantitative real time‐polymerase chain reaction
SD: standard deviation
SOD: superoxide dismutase
TBARS: thiobarbituric acid reactive substances
TG: triglyceride
T‐UDCA: tauro‐UDCA
UDCA: ursodeoxycholic acid
UPR: unfolded protein response
vWAT: visceral white adipose tissue

**Key Points**

- UDCA induces UPR signalling pathways via up‐regulation of *CHOP* and *GRP78* in NAFLD/NASH livers.
- Despite induced pro‐apoptotic *CHOP* expression, gene expression of hepatic pro‐apoptotic markers remains unchanged after UDCA.
- UDCA decreases pro‐apoptotic vesicle‐free miR‐34a levels in serum.
- Oxidative stress indicators are similar in UDCA treated and untreated morbidly obese patients.

## 1 INTRODUCTION

Non‐alcoholic fatty liver disease (NAFLD) is a general term reflecting a broad spectrum of obesity related liver disorders ranging from simple steatosis, over non‐alcoholic steatohepatitis (NASH) to fibrosis/cirrhosis and liver cancer.^1^, ^2^ The hallmark of NAFLD is hepatic triglyceride (TG) and free cholesterol accumulation, accompanied by peripheral insulin resistance (IR) in dysfunctional skeletal muscle and adipose tissue.^3^ Although obesity and consequently hepatic lipid deposition were considered as main disease triggers, variability in disease severity and outcome suggest that more complex, yet unknown mechanisms are involved.

The endoplasmic reticulum (ER) is the major site of protein biosynthesis and serves as cellular checkpoint for protein quality control.^4^ When misfolded proteins exceed ER folding capacity, the unfolded protein response (UPR) is activated via the three transmembrane stress sensors IRE1alpha, PERK and ATF6. Notably, obesity is linked to increased ER stress, representing another trigger of IR and diabetes.^5^ In addition, ER stress promotes apoptosis, a detrimental factor in NAFLD pathogenesis,^6^ which is mediated via *CHOP*, a *PERK* downstream target.^7^ Besides dysregulation of ER signalling and UPR, mitochondrial dysfunction triggering oxidative stress has been associated with obesity and IR in various tissues.^8^, ^9^ Mitochondria generate energy in the form of ATP via oxidative phosphorylation of nutrients such as free fatty acids.^10^ During obesity, in a state of oversupply of nutritional substrates and calories, reactive oxygen species (ROS) are formed. ROS are toxic by‐products of oxidative phosphorylation/beta‐oxidation and damage mitochondrial and cellular DNA, lipids and proteins.^8^ While obese humans with or without steatosis showed higher maximal respiration rates, NASH was associated with increased mitochondrial mass, leaking activity and hepatic oxidative stress.^11^ Therefore, ROS overproduction and thereof resulting oxidative stress are important players in NAFLD development and disease progression to NASH.^9^, ^12^, ^13^

Ursodeoxycholic acid (UDCA), an endogenous hydrophilic bile acid (BA), is currently in clinical use for the treatment of a wide range of liver diseases predominantly cholestatic disorders.^14^ Its cytoprotective effects have been ascribed to its hydrophilicity and its ability to reduce apoptotic signalling via the modulation of mitochondrial pathways.^15^ Additionally, the taurine‐conjugate of UDCA, T‐UDCA, has been reported to reduce ER stress markers in mouse liver,^16^, ^17^ but proved ineffective in human muscle and adipose tissue.^18^

In the present study, we aimed to uncover UDCA effects on (i) ER stress, (ii) oxidative stress and (iii) its potential anti‐apoptotic properties in liver samples of morbidly obese patients with NAFL/NASH. We show that UDCA induces one out of three UPR signalling pathways in the liver but also beneficially changes the UPR apoptotic threshold. Furthermore, we explore UDCA effects on hepatic oxidative stress signalling.

## 2 PATIENTS AND METHODS

### 2.1 Study population

This study included 40 well‐matched morbidly obese patients, recruited at Ersta Hospital, Stockholm, Sweden. Participants were equally randomized to UDCA treatment 20 mg/kg/d for 3 weeks (Ursofalk^®^, Dr. Falk, Freiburg, Germany; kind gift of MEDA, Stockholm, Sweden), or no treatment before bariatric surgery. The participants did not follow any specified diet and were instructed not to change their dietary habits during the study. Patients received UDCA until the evening before surgery. Blood sampling was performed in fasted state at 8:00 am, when liver and visceral white adipose tissue samples were taken. No day 21 blood was taken in the control group. Out of 40 patients, 19 finished in the UDCA and 18 in the control groups. Three drop‐outs were because of diarrhoea (UDCA group), pregnancy and minor intraoperative bleeding (control group). Detailed demographics have been reported before and show no significant difference in age, gender and body mass index.^19^

All participants provided written informed consent. The study protocol was approved by the Ethics Committee at Karolinska Institutet (Dnr 2008/2:3) and the Swedish Medical Products agency (EudraCT 2007‐005531‐28).

### 2.2 RNA expression analysis

Total RNA isolation from liver, complementary DNA synthesis, quantitative real‐time reverse transcription polymerase chain reaction (qRT‐PCR) and messenger RNA (mRNA) expression analysis was performed as previously described.^20^ mRNA expression levels were normalized to 18S. The housekeeping gene did not vary between the groups. micro‐RNA (miR) analysis via qRT‐PCR was performed as previously described.^21^ Serum exosome and RNA isolations were performed using the miRCURY Exosome Isolation Kit and miRCURY RNA Isolation Kit, respectively, according to the manufacturer's instructions (Exigon Life Sciences, Denmark).

### 2.3 Protein extraction and immunoblotting

Protein extraction and analysis via immunoblotting was performed as previously described.^22^ Antibodies were detected via commercial kits (Pierce ECL Plus Western Blotting Substrate, Thermo Scientific, USA). ImageJ (http.//imagej.nih.gov/ij/index.html) was used to quantify protein expression. Signals were normalized to beta‐actin or the corresponding phosphorylated or acetylated protein.

### 2.4 Lipid peroxidation assay

Thiobarbituric acid reactive substances (TBARS) were determined as previously described.^23^

### 2.5 Statistical analysis

Data are expressed as mean values ± standard deviation (SD). Differences were calculated with Mann**–**Whitney‐*U* Test analysing unequally distributed parameters using the SigmaStat^®^ statistic program (Jandel Scientific, San Rafael, CA, USA). A P value of <.05 was considered significant.

## 3 RESULTS

### 3.1 Fatty liver disease characteristics

**Figure 1:**
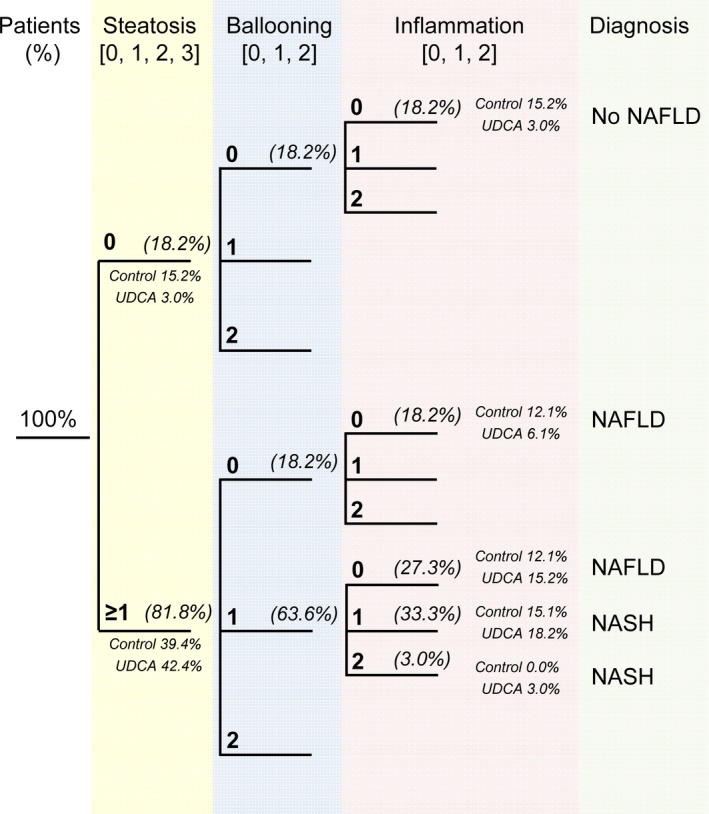
Diagnostic algorithm for the diagnosis of NAFLD/NASH. Patient numbers in (%) and according patient number per group (Control/UDCA) in (%)

To assess the stage of liver disease, liver biopsies of patients were classified according to histological criteria described by Bedossa et al^24^ by a pathologist (M.H.). In total, 18.2% patients were diagnosed no NAFLD (control 15.2%; UDCA 3.0%), 45.5% NAFLD (control 24.2%; UDCA 21.3%) and 36.3% were classified as NASH (control 15.1%; UDCA 21.2%) (Figure 1). Data on serum biochemistry further characterizing liver enzymes of UDCA treated and untreated patients can be found in Mueller et al^19^.

### 3.2 UDCA increased markers of endoplasmic reticulum stress

**Figure 2:**
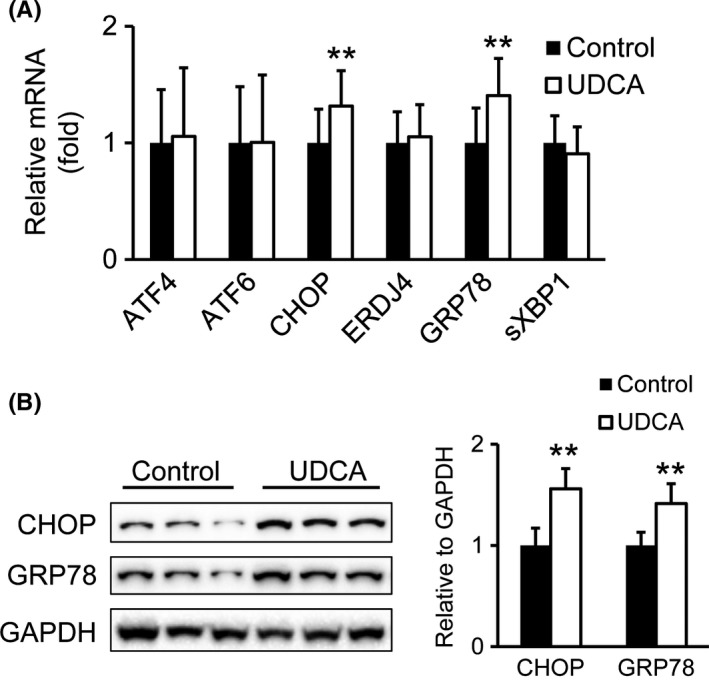
UDCA induces hepatic ER stress markers. (A) mRNA analysis of hepatic ER stress markers. Control: n = 18; UDCA: n = 19. (B) Protein levels of CHOP and GRP78. Representative Western blots are shown. Signal intensities were normalized to GAPDH. Control: n = 7; UDCA: n = 6. Mean values ± SD are expressed for all data. **P ≤ .01 vs control group

Indicators of ER stress were examined in liver and visceral white adipose tissue (vWAT) after UDCA treatment. While UDCA did not change ER‐stress markers in vWAT (data not shown), mRNA and protein expression levels of ER stress indicators such as *CHOP* and *GRP78* were elevated in patients after UDCA treatment compared to controls (Figure 2A,B). In contrast, other ER stress markers namely *ATF4, ATF6, ERDJ4* and *sXBP1*, were unchanged on mRNA level (Figure 2A).

**Figure 3:**
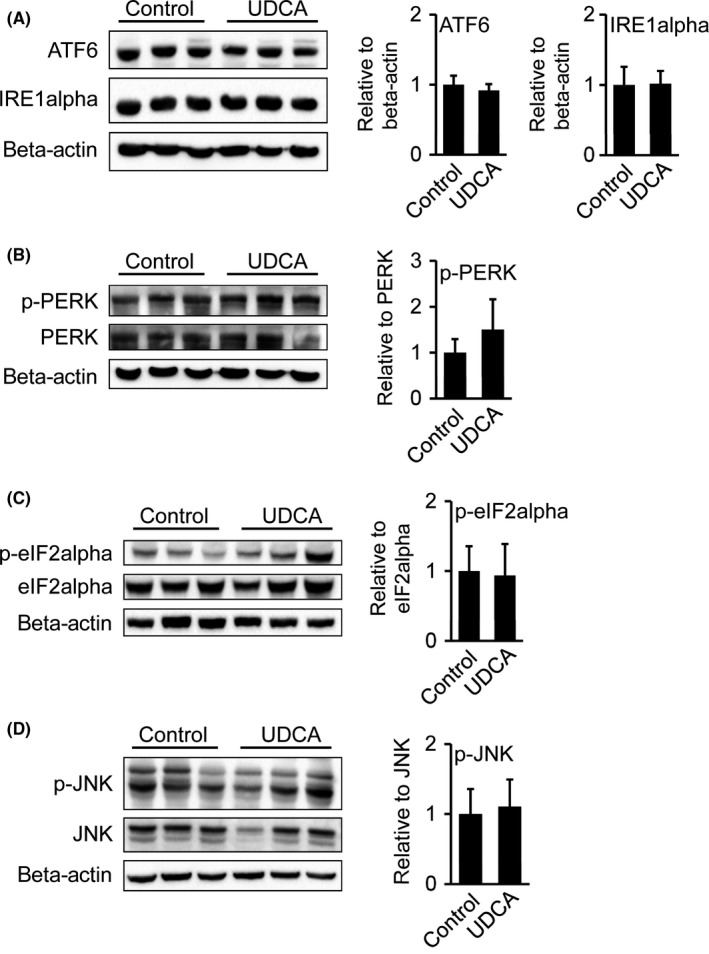
Protein levels of hepatic ER stress regulators remain unchanged after UDCA. (A) Protein levels of ER stress regulators ATF6, IRE1aplha. Signal intensities were normalized to beta‐actin. (B, C, D) Protein levels of phosphorylated PERK and total PERK, phosphorylated eIF2alpha and total eIF2alpha, phosphorylated JNK and total JNK. Representative Western blots are shown. Signal intensities were normalized to beta‐actin and ratio of phosphorylated vs total protein was calculated. Control: n = 7; UDCA: n = 6. Mean values ± SD are expressed for all data

Phosphorylation is an important signal transducer in ER stress.^25^ To further investigate whether UDCA interferes with hepatic ER stress, we analysed protein levels and/or their phosphorylation status of the three transmembrane effector proteins triggering the UPR as well as their downstream targets *ATF6*,* IRE1alpha*,* PERK* and *JNK*. Neither protein levels of the ER stress sensor ATF6 nor IRE1alpha were increased in liver homogenates of UDCA treated patients compared to untreated controls (Figure 3A). The ratio of phosphorylated to total PERK was moderately but not significantly increased in liver preparations of UDCA treated patients (Figure 3B). This is in accordance with significantly elevated *CHOP* expression (Figure 2A,B), a *PERK* downstream target.^7^ Additionally, the ratio of phosphorylated to total eIF2alpha, another *PERK* target,^7^ was determined, but did not differ between the groups (Figure 3C).

The association of JNK activation to ER stress signalling^7^ prompted us to measure JNK‐phosphorylation in liver homogenates. During ER stress, there are two potential JNK‐activation routes: via IRE1alpha or the metabolic inflammasome harbouring eIF2alpha.^26^, ^27^ However, phosphorylation ratio and total protein levels of JNK were similar comparing UDCA treated and untreated patients (Figure 3D).

Taken together, despite elevated mRNA levels of the ER stress indicators *CHOP* and *GRP78*, the PERK ratio of protein phosphorylation to total protein was only moderately but not significantly increased.

### 3.3 CHOP activation as a potential pro‐apoptotic factor after UDCA treatment

**Figure 4:**
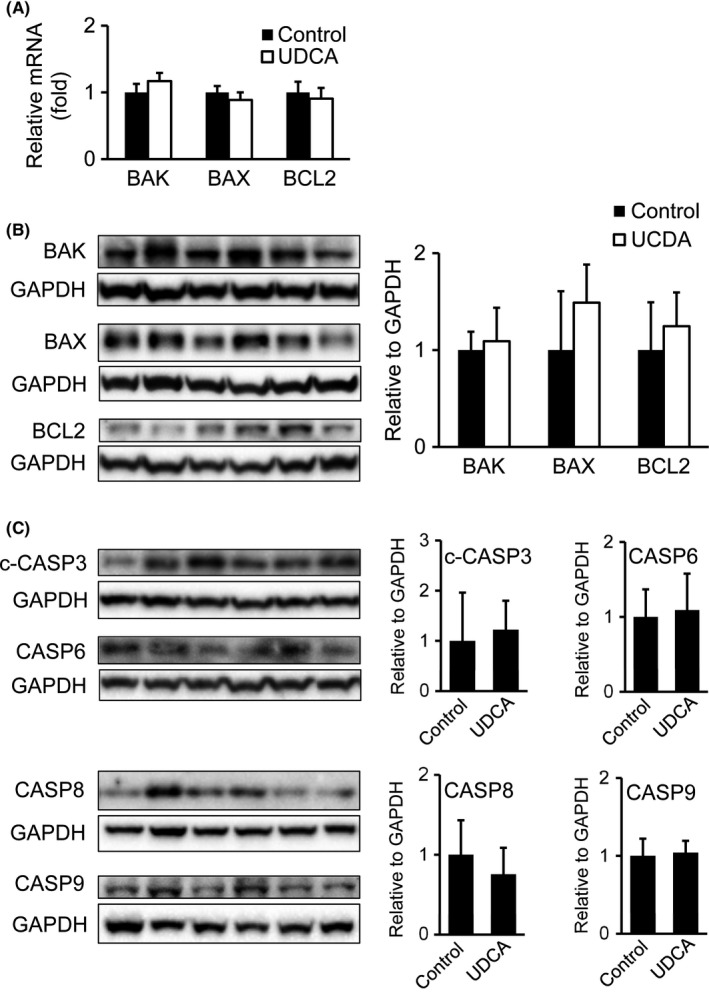
UDCA shows minor impact on hepatic apoptosis markers, cell survival markers and caspase signalling. (A) mRNA analysis of apoptosis and cell survival markers in liver of UDCA treated and untreated patients. Control: n = 18; UDCA: n = 19. (B) Immunoblotting of BAK, BAX and BCL2. Signal intensities were normalized to GAPDH. Representative Western blots are shown. Control: n = 7; UDCA: n = 6. (C) Protein expression of cleaved‐CASP3, CASP6, CASP8 and CASP9 via Western blotting. Signal intensities were normalized to GAPDH. Representative Western blots are shown. Control: n = 7; UDCA: n = 6. Mean values ± SD are expressed for all data

ER‐stress mediated apoptosis is largely driven by *CHOP*. It has been shown that this transcription factor induces the expression of several pro‐apoptotic genes.^28^, ^29^ Considering UDCA's established cytoprotective properties,^17^ elevated *CHOP* mRNA levels after UDCA treatment, prompted us to further evaluate UDCA effects on cell survival/apoptosis in morbidly obese patients. mRNA and protein expression of the pro‐ and anti‐apoptotic genes *BAK*,* BAX* and *BCL2,* respectively, were similar between the groups (Figure 4A,B). Moreover protein levels of additional effectors and initiators of apoptosis such as cleaved‐CASP3, CASP6 and CASP8, CASP9, were analysed but did not show any changes after UDCA treatment (Figure 4C). Absent induction of apoptosis markers (downstream of *CHOP*) and caspase signalling suggests that UDCA possibly evens out *CHOP's* pro‐apoptotic actions.

### 3.4 Serum miR‐34a levels decreased after UDCA, though hepatic miR‐34a/SIRT/p53 signalling pathway remains unchanged

**Figure 5:**
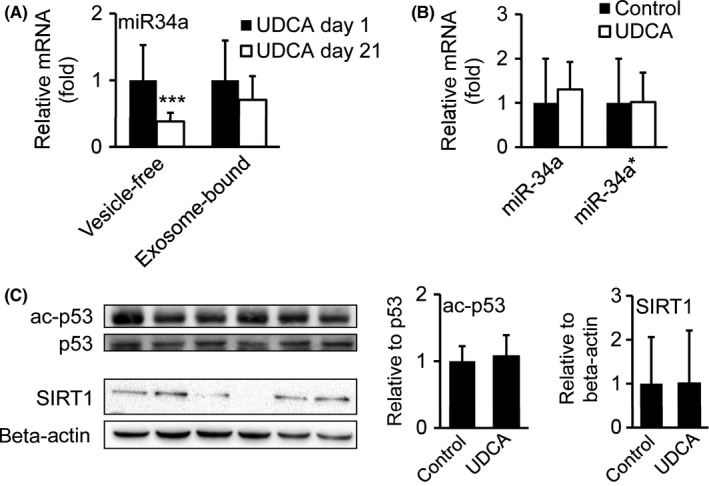
UDCA decreases pro‐apoptotic miR‐34a levels in vesicle‐free serum fractions but not in liver tissue of morbidly obese patients. qRT‐PCR analysis of miR‐34a in vesicle‐free and exosome‐bound serum fractions of UDCA treated patients comparing day 1 (before treatment) and day 21 (end of treatment). UDCA day 1: n = 14; UDCA day 21: n = 14. (B) qRT‐PCR analysis of miR‐34a forward strand and miR‐34a reverse strand (miR‐34a*) in liver tissue of UDCA treated and untreated patients. Control: n = 18; UDCA: n = 19. (C) Immunoblotting of acetylated p53, total p53 and SIRT1. Signal intensities were normalized to beta‐actin and ratio of acetylated vs total protein was calculated. Representative Western blots are shown. Control: n = 7; UDCA: n = 6. Mean values ± SD are expressed for all data

Besides pro‐apoptotic signalling via *CHOP* and *BAK*/*BAX*, apoptosis can also be mediated via the miR‐34a/SIRT/p53 signalling pathway.^30^ Indeed, UDCA has been proven beneficial in the modulation of the miR‐34a/SIRT/p53 pathway and liver cell apoptosis.^30^ Thus, we examined miR‐34a expression in vesicle‐free and exosome‐bound serum fractions before and after UDCA treatment (Figure 5A) as well as in liver tissue from UDCA treated patients and controls (Figure 5B). While miR‐34a expression was exclusively and markedly decreased in the vesicle‐free serum fraction after UDCA, miR‐34a levels in the exosome‐bound serum fraction remained unaffected (Figure 5A). Neither hepatic miR‐34a forward nor miR‐34a reverse strand (miR‐34a*) were changed after UDCA treatment (Figure 5B). Because of possible transactivation properties of miR‐34a by p53,^31^ we further delineated potential UDCA actions on hepatic miR‐34a/p53/SIRT1‐pathway. Protein ratio of acetylated to total p53, indicating protein activity,^32^ and levels of total SIRT1 were unaltered (Figure 5C). Even though a decrease in miR‐34a levels in vesicle‐free serum fractions indicates a potential suppression of pro‐apoptotic signal transduction in serum after UDCA treatment, data suggest minor impact of UDCA at the applied dosage and treatment period on liver tissue in morbidly obese patients.

### 3.5 Unaltered oxidative stress parameters after UDCA

**Figure 6:**
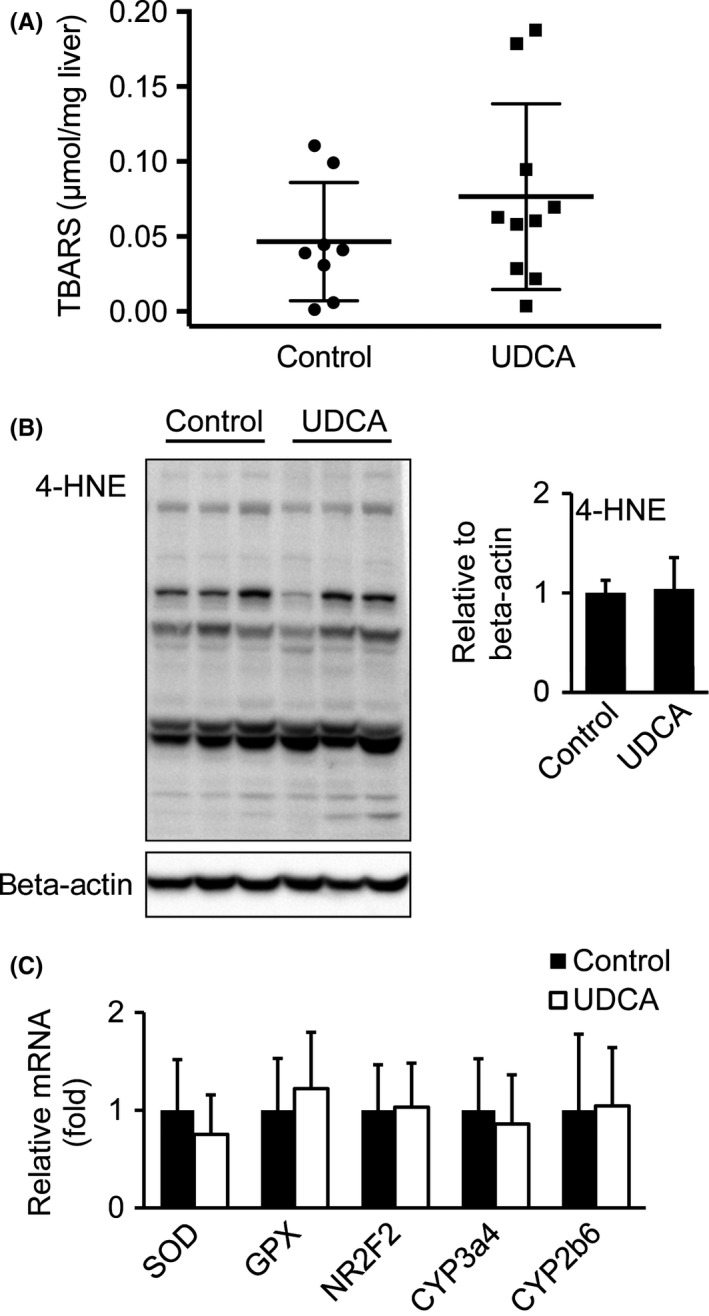
Oxidative stress levels remain unchanged after UDCA treatment. (A) Thiobarbituric acid reactive substances (TBARS) levels were measured from liver homogenates by TBA assay as an indicator for lipid peroxidation. Control: n = 8; UDCA: n = 10. (B) Immunoblotting of 4‐HNE‐conjugated protein levels. Signal intensities were normalized to beta‐actin. Control: n = 7; UDCA: n = 6. (C) Hepatic mRNA expression of enzymes (*SOD, GPX, CYP3a4, CYP2b6*) and transcription factor (*NR2F2*) as oxidative stress indicators. Control: n = 18; UDCA: n = 19. Mean values ± SD are expressed for all data

Since oxidative stress has been implicated in the progression of NASH and may represent a valid therapeutic target,^33^ oxidative stress parameters were determined in liver homogenates. TBARS, a lipid peroxidation product,^34^ was unchanged after UDCA treatment (Figure 6A). Additionally, immunoblotting of 4‐hydroxynonenal (4‐HNE), another peroxidation product forming stable adducts with proteins,^35^ revealed similar adduct formation levels in UDCA treated and untreated groups (Figure 6B). Consistent with unchanged formation of peroxidation products, hepatic mRNA expression of oxidative stress markers, such as superoxide dismutase (*SOD*), the enzyme converting superoxide to hydrogen peroxide and the *SOD*‐downstream enzyme glutathione peroxidase (*GPX*), metabolizing hydrogen peroxide to non‐toxic H_2_O, remained unaffected after UDCA treatment (Figure 6C). Expression of *NR2F2*, a transcription factor, inducing a cascade of oxidative stress response genes,^36^ as well as mRNA levels of *CYP3a4* and *CYP2b6* were stable between the groups (Figure 6C).

## 4 DISCUSSION

In this study, analysis of liver tissue obtained during bariatric surgery from short‐term UDCA treated and untreated morbidly obese NAFLD‐patients revealed increased UPR signalling, changes in circulating miR‐34a levels and minor effects on pro/anti‐apoptotic signalling as well as oxidative stress in response to UDCA treatment. We provide an elaborate analysis of a human study depicting the impact of BA treatment on apoptosis and stress signalling pathways in morbid obesity and thereby give relevant insight in the efficacy of short‐term UDCA treatment and development of future bile acid‐based therapies in human NAFLD.

Several studies have focused on UPR activation and its association with NAFLD development and NASH.^37^ Furthermore, TUDCA, the taurine conjugated form of UDCA, showed a considerable decrease in ER stress parameters in cultured cells as well as in a mouse model of type 2 diabetes. These effects have been attributed to its properties as chemical chaperone.^16^ Interestingly and in strong contrast to TUDCA, we found that in NAFLD‐patients highly enriched short‐term UDCA enhances hepatic ER stress via induction of ER stress markers such as *CHOP* and *GRP78*. Moreover, ER stress elicits a broader metabolic function: *XBP1* functions as key mediator of the unfolded protein response and directly regulates, though in an ER stress response‐unrelated manner, pro‐lipogenic genes in the liver.^38^ Furthermore, it is established that ER stress induces lipogenesis via *SREBP1c* resulting in hepatic lipid accumulation.^39^ In line with this observation by Lee et al, elevated ER stress marker expression was paralleled by increased SAF‐scoring, stearoyl‐CoA protein levels, a lipogenic trigger, and hepatic triglyceride accumulation in the UDCA treated group of this study cohort, as previously reported.^19^ At first glance, one could suggest that short‐term UDCA treatment stimulated hepatic lipid accumulation via the activation of ER stress signalling. However, taking former results of this study cohort into consideration, the metabolic situation may be far more complex. UDCA has low affinity for the nuclear farnesoid X receptor (*FXR*), which is known for its anti‐lipogenic properties.^40^ Overabundance of UDCA, constituting close to 90% of the BA pool in the treatment group, rendered *FXR* activation to be diminished.^19^ As a consequence, higher concentrations of TG were stored in the liver. Excessive hepatic lipid loads in UDCA treated patients, in turn, led to a dysfunctional lipid management and initiation of the UPR as an attempt to restore ER homeostasis.

Besides the knowledge of chronic ER stress present in liver and adipose tissue in mouse models of obesity and obese humans,^28^, ^41^ recently, phosphorylation of eIF2alpha, an UPR signalling protein, was shown to be elevated in the context of NAFL and NASH in humans.^37^ Considering TUDCA's beneficial effects on ER stress,^28^ one would also expect changes in protein content and/or phosphorylation status of ER stress mediating membrane proteins after treatment with unconjugated UDCA. However, it is possible that taurine conjugation, which is a minor form of conjugate in humans, is required to alleviate ER stress because unconjugated UDCA over a period of 3 weeks was not sufficient to drastically impact on protein expression or phosphorylation in NAFL/NASH. Nevertheless, it is conceivable that despite elevated *CHOP* and *GRP78* levels, likely stimulated via hepatic lipid accumulation, UDCA treatment is capable of preventing further derangements in ER signalling.

Considering that apoptosis is a detrimental factor in NAFLD,^6^ it is noteworthy that *CHOP*, also known as growth arrest‐ and DNA damage‐inducible gene 153, drives ER stress mediated apoptosis.^42^ Despite elevated *CHOP* expression, unchanged apoptosis or cell survival markers and caspase signalling further support the concept that UDCA may be beneficial via changing the apoptotic threshold and protecting hepatocytes against more pronounced cell death. However, this needs to be confirmed by future larger long‐term studies.

The miR34a/SIRT1/p53‐pathway represents an additional route, via which hepatocyte apoptosis is induced in NAFLD. In rat primary hepatocytes, UDCA achieved profound downregulation of pro‐apoptotic miR‐34a/SIRT1/p53‐signalling.^30^ Analysis of liver tissue indicated that UDCA did not influence miR‐34a, p53 or SIRT1 expression in this rather small study cohort. We assume that liver specific UDCA effects on miR‐34a/SIRT1/p53‐signalling may underlie time‐ and dose‐dependent kinetics. However, we show that UDCA decreased vesicle‐free miR‐34a in serum. While exosome‐bound miRNAs are considered relatively stable and therefore are in evaluation as potential disease biomarkers, vesicle‐free miRNAs are quickly targeted for degradation.^43^ The mechanism of accelerated vesicle‐free miR‐34a degradation after UDCA treatment requires further elucidation.

Oxidative stress occurs because of excessive beta‐oxidation thereby provoking unbalanced ratios of pro‐ and antioxidants. According to observations over the past decades, there is increased incidence of systemic and hepatic oxidative stress in patients with NAFL and NASH.^13^, ^44^, ^45^ In the present short‐term high‐dose study, we could not identify marked differences in oxidative stress status in UDCA treated patients. Certainly, it would be of interest to compare our data with a 2‐year trial in NASH patients receiving UDCA and antioxidant treatment.^46^ However, Dufour et al focused on serum parameters and histology. Therefore, we cannot provide evidence whether absent beneficial UDCA effects on oxidative stress parameters and markers are time dependent.

Some limitations of our study deserve further discussion: The study lacks a placebo control and biopsies were, for ethical reasons, obtained only after UDCA therapy, which did not allow paired sample testing. Because of restricted biopsy material availability, distinct results were assessed by mRNA analysis only, however, key findings were investigated on protein level and were also supported by biochemical methods. The pooling of all patients into the two studied groups (UDCA‐treated and ‐untreated), although necessary given small numbers of patients in some particular scores, carries the limitation of comparing patients at different stages of disease within the same group. The data of this short‐term study need to be interpreted with caution since comparison with long‐term effects of UDCA may be difficult.

In conclusion, this prospective pharmacodynamic study in morbidly obese patients delivered additional insights into the therapeutic efficacy and potential limitations of UDCA in NAFLD. UDCA effects ‐ the good and the bad ‐ have raised the paradox that treatment induces hepatic UPR via *GRP78* and the pro‐apoptotic factor *CHOP* on the one hand, and on the other shows the strong potential to increase apoptotic thresholds. We searched for evidence of UDCA mediated improvement of aberrant oxidative status but failed to demonstrate any efficacy. The enthusiasm for the possibility of treating NAFLD‐patients with UDCA has waned but might have created the necessary incentive to further develop other bile acid based therapies.

## CONFLICT OF INTEREST

The authors do not have any disclosures to report.
